# An adenosine A3 receptor agonist inhibits DSS-induced colitis in mice through modulation of the NF-κB signaling pathway

**DOI:** 10.1038/srep09047

**Published:** 2015-03-12

**Authors:** Tianhua Ren, Ting Tian, Xiao Feng, Shicai Ye, Hao Wang, Weiyun Wu, Yumei Qiu, Caiyuan Yu, Yanting He, Juncheng Zeng, Junwei Cen, Yu Zhou

**Affiliations:** 1 Department of Gastroenterology, The Affiliated Hospital of Guangdong Medical College, No. 57 South Renmin Avenue, Zhanjiang 524001, China

## Abstract

The role of the adenosine A3 receptor (A3AR) in experimental colitis is controversial. The A3AR agonist N^6^-(3-iodobenzyl)adenosine-5'-N-methyluronamide (IB-MECA) has been shown to have a clinical benefit, although studies in A3AR-deficient mice suggest a pro-inflammatory role. However, there are no studies on the effect of 2-Cl-IB-MECA and the molecular mechanism of action of A3AR in murine colitis models in vivo. Is it the same as that observed in vitro? The interaction between 2-CL-IB-MECA and A3AR in a murine colitis model and the signaling pathways associated with this interaction remain unclear. Here we demonstrate a role for the NF-κB signaling pathway and its effect on modifying the activity of proinflammatory factors in A3AR-mediated biological processes. Our results demonstrated that A3AR activation possessed marked effects on experimental colitis through the NF-κB signaling pathway.

Inflammatory bowel disease (IBD), a chronic and relapsing inflammation of the gastrointestinal tract, affects millions of people worldwide. The chronic mucosal inflammation in IBD is caused by hyperactivation of effector immune cells, which produce high levels of pro-inflammatory cytokines like tumor necrosis factor-alpha (TNF-α), interleukin-1beta (IL-1β), and interferon-gamma (IFN-γ), thereby resulting in colonic tissue damage. Nuclear factor-kappa B (NF-κB) was identified as one of the key regulators in this immunological setting^1^,^2^,^3^. Its activation is markedly induced in IBD patients, and through its ability to promote the expression of various pro-inflammatory genes, NF-κB strongly influences the course of mucosal inflammation. Proinflammatory cytokines and bacterial pathogens activate NF-κB, mostly through inhibitory kappa B (IκB) kinase-dependent phosphorylation and degradation of IκB proteins. Inhibition of NF-κB activation has been suggested as an anti-inflammatory strategy in IBD.

Dextran sulfate sodium (DSS) is commonly used in rodent models to chemically induce intestinal inflammation^4^,^5^. DSS-induced colitis is characterized by weight loss, bloody diarrhea, epithelial cell damage, and immune cell infiltration, as well as an increased production of inflammatory mediators, including TNF-α, IL-1β, etc. NF-κB was activated to induce such inflammatory mediators^6^,^7^. Colitis may result from DSS toxicity to colonic epithelial cells.

Adenosine, a purine nucleoside, is released from metabolically active cells into extracellular space and plays an important role in various pathophysiological processes. Adenosine regulates many biological responses, including inflammation, by interaction with its receptors such as A1, A2A, A2B, and A3^8^,^9^. Adenosine A1, A2, and A3 receptor proteins or mRNA are present in the rodent and human intestinal tract^10^,^11^,^12^,^13^. High levels of extracellular adenosine may lead to the activation of the A2A receptor to suppress the chronic inflammation in IBD models^8^. Moreover, activating A2B receptor on intestinal epithelial cells can augment IL-6 production and increase neutrophil activation in human colitis. Additionally, the administration of a selective A2B antagonist ATL-801 can inhibit the above pathological processes^9^. However, the function of A3AR is unknown.

Adenosine A3 receptor (A3AR) belongs to the Gi/Gq-coupled receptor family. Stimulation of A3AR inhibits adenylate cyclase activity, activates phospholipase C, and triggers calcium mobilization, leading to modulation of signaling pathways such as WNT, PI3K/AKT, and NF-κB^14^,^15^.

Inflammation can drive A3AR expression and induce up-regulation in peripheral blood mononuclear cells in rheumatoid arthritis and Crohn's disease^16^. The expression of A3AR in colonic crypt epithelial cells was reduced significantly in DSS-induced murine colitis^17^. The A3AR agonist N^6^-(3-iodobenzyl)adenosine-5'-N-methyluronamide(IB-MECA) was shown to ameliorate inflammation in two different mouse models of colitis, including DSS-induced colitis and spontaneous colitis found in IL-10-deficient mice^18^, whereas Ren et al. recently reported that A3AR-deficient mice were less susceptible to DSS-induced colitis^17^. The inflammatory mechanisms controlling these phenotypes in A3R-knockout mice have yet to be fully elucidated. Therefore, the role of A3AR in gut inflammation needs further clarification.

The effects of IB-MECA occur at high doses ranging from 1–3 mg/Kg body weight and may not necessarily be restricted to actions at A3AR as both high-affinity (A1, A2A) and low-affinity (A2B and A3AR) receptors may be activated by oral A3AR drugs^17^. The pharmacology is complex since all four adenosine receptors are potential therapeutic targets in experimental models of IBD, thus agonist drugs (A1, A2A, A3) and antagonist drugs (A2B) may have protective or therapeutic effects in colitis models or other inflammatory diseases^10^,^11^,^17^,^18^,^19^,^20^.

A synthetic, highly-selective agonist for A3AR was introduced for the treatment of inflammation. In vitro, 2-chloro-N^6^-(3-iodobenzyl)adenosine-5'-N-methylcarboxamide (2-Cl-IB-MECA) has been shown to be a potent A3AR agonist with a 2,500- and 1,400-fold selectivity for A3AR versus A1AR and A2AR, respectively^21^,^22^. The high selectivity of 2-Cl-IB-MECA enabled us to study A3AR-mediated effects in inflammation, without the interference or blockade of the effects of other adenosine receptor subtypes^14^. In vivo studies have shown that 2-Cl-IB-MECA reduces ischemia/reperfusion injury in mice by specifically activating A3AR^23^. 2-Cl-IB-MECA has been used to study the control of enteric neuromuscular functions by A3AR in the normal rat distal colon and in experimental bowel inflammation^12^. However, to date, no studies have elucidated the effect of 2-Cl-IB-MECA in murine colitis models in vivo. Further studies on the molecular mechanism of action of A3AR and effects of the A3AR agonist 2-Cl-IB-MECA on IBD are needed.

Thus, this study utilized the DSS colitis mouse model to investigate further the role of the selective A3AR agonist 2-Cl-IB-MECA in the development of murine colitis in vivo and the potential mechanism underlying its effects. We hypothesize that the A3AR agonist 2-Cl-IB-MECA attenuates the development of DSS colitis associated with the NF-κB signaling pathway.

## Results

### 2-Cl-IB-MECA attenuates clinical activity of DSS-induced murine colitis

**Figure 1:**
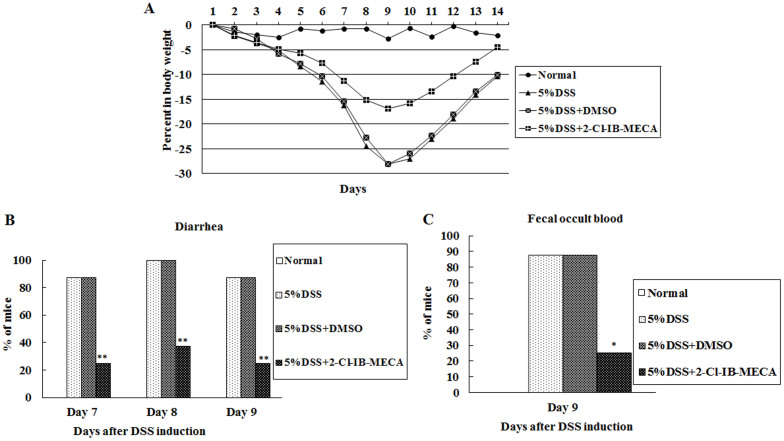
2-Cl-IB-MECA attenuates clinical activity of DSS-induced murine colitis. (A) DSS-induced weight loss is ameliorated by 2-Cl-IB-MECA administration. In animals receiving 5% DSS, maximum weight loss was significantly reduced to 17% (with 2-Cl-IB-MECA treatment) from 28% (with DMSO treatment). After 9 days, 2-Cl-IB-MECA-treated mice recovered faster. ANOVA between all groups, *P* < 0.01; post-tests, 5% DSS + 2-Cl-IB-MECA versus 5% DSS + DMSO, *P* < 0.01; 5% DSS + DMSO versus 5% DSS, *P* > 0.05. 2-Cl-IB-MECA administration reduced diarrhea and fecal occult blood. (B) 5% DSS colitis animals mostly develop diarrhea on days 7–9, whereas 2-Cl-IB-MECA-treated animals induced with 5% DSS exhibited reduced diarrhea (**chi-square test comparing incidence of diarrhea between vehicle-treated and 2-Cl-IB-MECA-treated mice induced with 5% DSS, *P* < 0.01). (C) 2-Cl-IB-MECA-treated mice have a lower incidence of occult blood than did vehicle-treated animals on day 9 after 5% DSS induction (*chi-square test, DMSO versus 2-Cl-IB-MECA, *P* < 0.05).

Oral administration of 2-Cl-IB-MECA significantly reduced clinical activity of DSS-induced murine colitis. No significant difference was observed between vehicle-treated mice with DSS colitis and mice with DSS colitis alone (Fig. 1).

Clinical activity was evaluated using the parameters of weight loss, stool consistency, and the guaiac fecal occult blood test. 2-Cl-IB-MECA reduced weight loss, diarrhea, and fecal occult blood (Fig. 1). After DSS induction, mice lose weight over time with a maximum of 28% body weight loss within 10 days. After withdrawal of DSS in the drinking water, animals began to recover and gain weight. At 5% DSS, 2-Cl-IB-MECA-treated mice lost 17% body weight compared to 28% in vehicle-treated mice and recovered faster than vehicle-treated mice (Fig. 1A). In 5% DSS colitis animals, most animals developed diarrhea on days 6–10. In contrast, 2-Cl-IB-MECA-treated mice induced with 5% DSS had reduced diarrhea on days 7–9 (Fig. 1B). The guaiac test was used to evaluate the presence of occult blood in stools of each animal separately. A positive guaiac test was observed in most animals on days 6–10 in mice induced with 5% DSS. However, 2-Cl-IB-MECA treatment had a lower incidence of occult blood than vehicle-treated mice on day 9 after DSS induction. In vehicle-treated mice, 87.5% of animals still had a positive guaiac test after 9 days as compared to 25% of animals in 2-Cl-IB-MECA-treated mice (Fig. 1C).

### 2-Cl-IB-MECA administration reduced inflammation and tissue injury, inhibited MPO activity, and decreased TNF-a and IL-1β expression in DSS-induced murine colitis

#### Histopathology and MPO Activity

We next evaluated the severity of acute murine colitis by blinded histological injury scoring in the distal colon. The histological examination of distal colons from DSS-induced colitis mice revealed inflammatory lesions that included ulceration, crypt damage, and infiltration of inflammatory cells consisting of macrophages, lymphocytes, and neutrophils.

**Figure 2:**
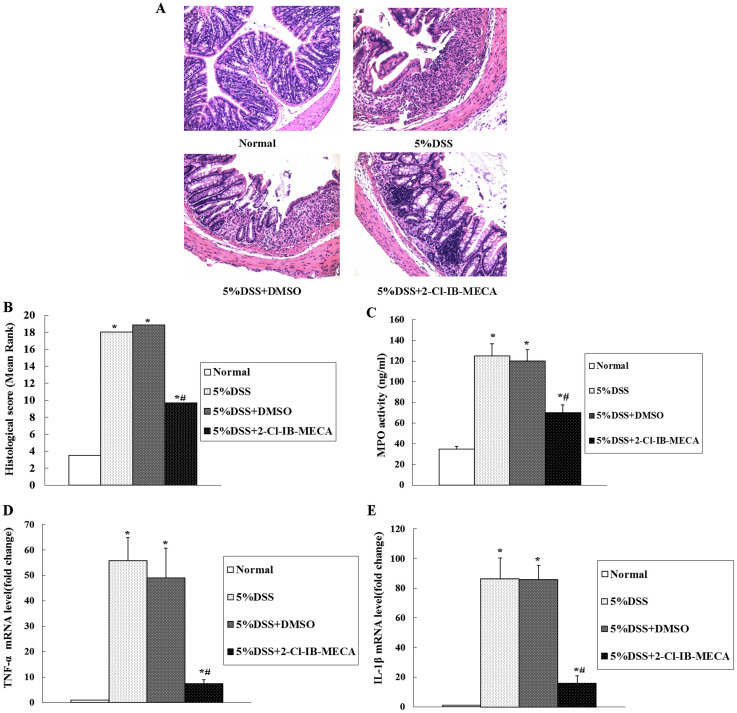
2-Cl-IB-MECA administration reduced inflammation and tissue injury, inhibited MPO activity, and decreased TNF-a and IL-1β expression in DSS-induced murine colitis. (A) 2-Cl-IBMECA administration reduced H&E histopathology in murine DSS colitis. In normal mice, there was no active inflammation, crypt or surface epithelial damage, or ulceration. In mice treated with 5% DSS with or without DMSO, transmural inflammation, crypt damage, and ulceration were observed. In 5% DSS with 2-Cl-IB-MECA, focal active inflammation without crypt or surface epithelial damage or ulceration was observed. (B) 2-Cl-IB-MECA administration protects mice from gut inflammation and tissue injury. Aggregate histopathological scoring of inflammation, ulceration, and crypt damage indicates protection. A Mann–Whitney nonparametric U-test was used. (C) 2-Cl-IB-MECA inhibits MPO activity after DSS induction of colitis. MPO elevation in DSS-induced colitis is reduced by 2-Cl-IB-MECA administration. TNF-a (D) and IL-1β (E) mRNA were expressed at relatively low levels in the normal group. Induction of colitis with 5% DSS resulted in an increase in TNF-a and IL-1β mRNA expression. However, 2-Cl-IB-MECA significantly reduced DSS-induced TNF-a and IL-1β mRNA expression in colitis mice. No significant difference was observed between vehicle-treated mice with DSS colitis and mice with DSS colitis alone. Data are presented as mean ± SD of three independent experiments (*P < 0.01 compared with the normal group; #*P* < 0.05 compared with the DSS group).

In contrast, the administration of 2-Cl-IB-MECA markedly reduced the mucosal damage. Representative examples of H&E histopathology for 2-Cl-IB-MECA treated mice and vehicle treated mice induced with DSS are shown in Fig. 2A. Histological scoring showed that treatment with 2-Cl-IB-MECA significantly reduced the overall score relative to that of the vehicle-treated colitis mice (Fig. 2B). In addition to the clinical and histological severity, the level of MPO activity, as a parameter of neutrophil accumulation, was marginally detectable in the absence of DSS induction, but was increased in colons of mice with DSS-induced colitis (Fig. 2C). Treatment with 2-Cl-IB-MECA significantly suppressed the up-regulated MPO activity compared to vehicle-treated mice induced with DSS.

### 2-Cl-IB-MECA suppressed DSS-induced pro-inflammatory cytokine expression in murine colon epithelia

We investigated whether 2-Cl-IB-MECA administration alters the production of pro-inflammatory cytokines, including TNF-a and IL-1β, in colon epithelia of DSS colitis mice. As shown in Fig. 2D and E, TNF-a and IL-1β mRNA showed relatively low expression levels in the control groups without DSS. DSS induction resulted in an increased mRNA expression of TNF-a and IL-1β respectively (*P* < 0.01). No significant difference was observed between vehicle-treated mice with DSS colitis and mice with DSS colitis alone. However, 2-Cl-IB-MECA treatment significantly inhibited TNF-a and IL-1β expression (*P* < 0.05).

### 2-Cl-IB-MECA attenuated DSS-induced NF-κB signaling pathway activation in murine colon epithelia

Given that the previous studies showed that 2-Cl-IB-MECA could exert its anti-inflammatory effects by blocking NF-κB activation in some inflammatory diseases in vitro and in vivo, we investigated this signaling pathway in the DSS-colitis murine model to confirm its role in vivo.

### Location and qualitative expression of IκB-α, p-IκB-α, and NF-κB p65 in murine colon epithelia

**Figure 3:**
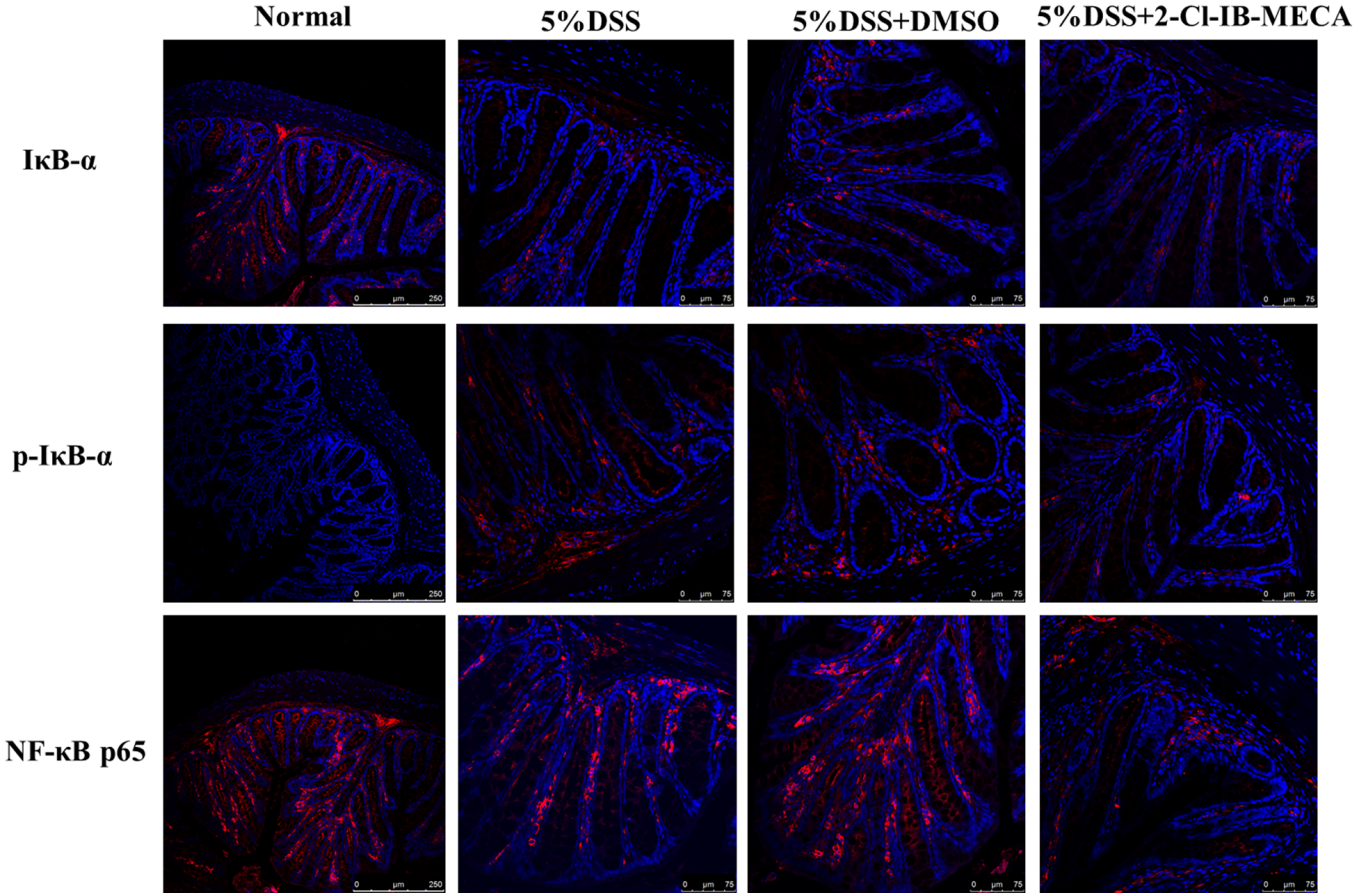
Location and qualitative expression of IκB-α, p-IκB-α and NF-κB p65 in murine colon epithelia. IF was performed using specific antibodies to label target proteins. NF-κB p65 was limited to the epithelial cytoplasm in the normal colon, and nuclear localization can be observed after DSS-induced colitis. However, a significant reduction in p65 nuclear translocation was seen in 2-Cl-IB-MECA-treated mice. There was a tendency for decreased IκB-α and increased p-IκB-α expression in the cytoplasm after DSS colitis, which was inhibited in the 2-Cl-IB-MECA-treated group. These results suggested that 2-Cl-IB-MECA inhibited DSS-induced phosphorylation of IκB-α and the nuclear translocation of NF-κB p65 in colonic epithelia.

To determine the involvement of NF-κB signaling pathway activity in the in vivo model of colitis, immunofluorescence was performed on colon sections of mice to verify the presence and distribution of IκB-α, p-IκB-α and NF-κB p65 in epithelia. The micrographs are shown in Fig. 3. In the DSS-induced colitis group, impaired IκB-α and increased expression of p-IκB-α in cytoplasm can be observed. Meanwhile, in normal colons without DSS induction, NF-κB p65 was mainly located in the cytoplasm and absent from the nucleus. DSS colitis induction increased NF-κB p65 activity and translocation into nuclei, as a shift of NF-κB p65 towards the nuclear area can be observed. Treatment with 2-Cl-IB-MECA suppressed the phosphorylation of IκB-α in epithelia cytoplasm and attenuated NF-κB p65 nuclear translocation.

### Quantitative expression of IκB-α, p-IκB-α, and NF-κB p65 in murine colon epithelia

**Figure 4:**
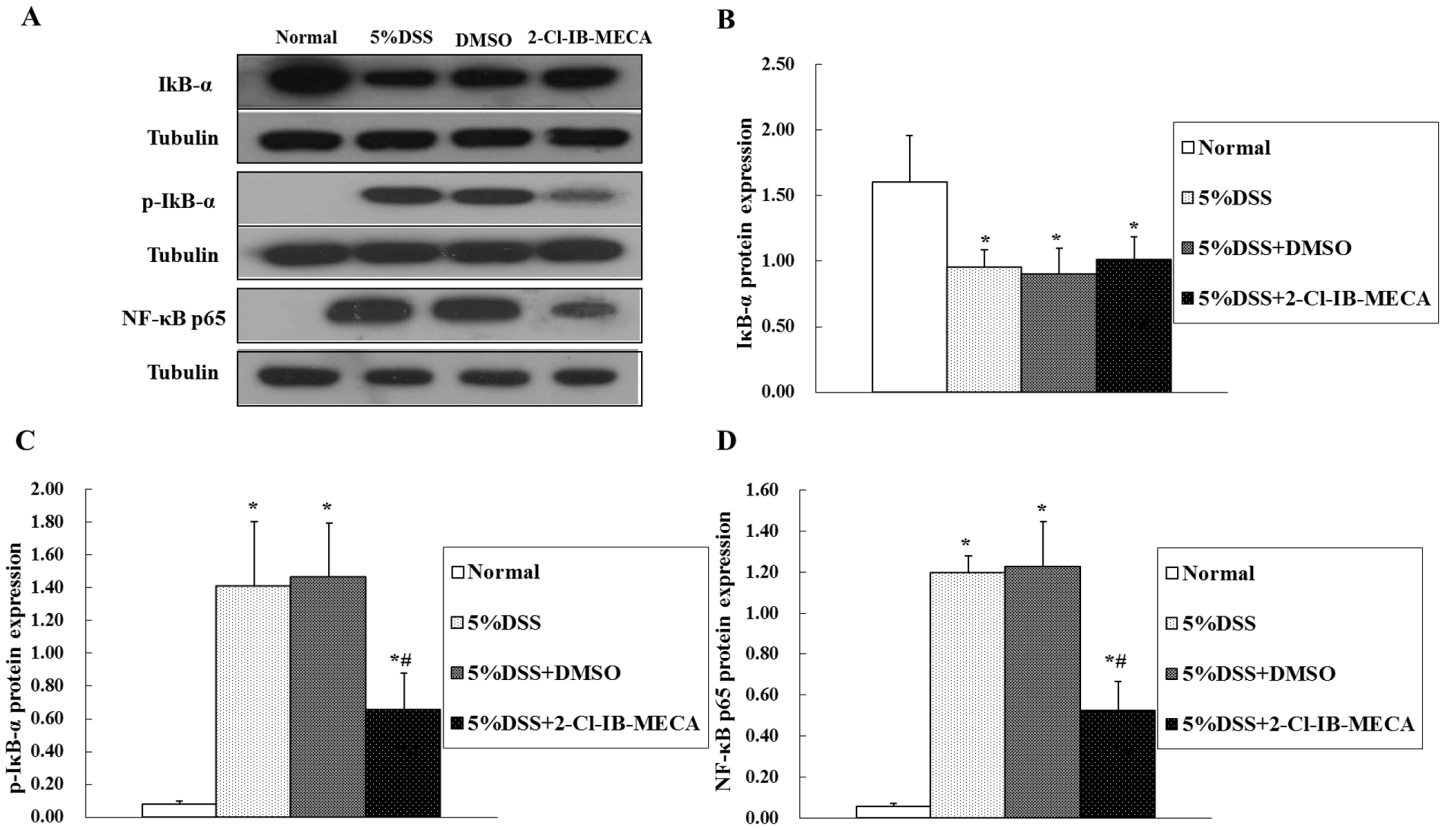
Effect of 2-Cl-IB-MECA on DSS-induced NF-κB p65 signaling pathway activation in murine colon epithelia. Cytosolic and nuclear proteins were extracted separately and analyzed by western blot. DSS administration resulted in a reduction in IκB-α (A, B) and an increase in p-IκB-α (A,C) in the cytoplasm, and an increase of NF-κB p65 (A,D) in nuclear extracts from murine colon epithelia, indicating DSS colitis induced phosphorylation and degradation of IκB-α in the cytoplasm and NF-κB p65 translocation from the cytoplasm to the nucleus. Compared with the DSS colitis group, 2-Cl-IB-MECA treatment attenuated the expression of p-IκB-α in the cytoplasm and decreased the expression of NF-κB p65 in the nucleus, suggesting 2-CL-IB-MECA inhibited DSS colitis and induced phosphorylation of IκB-α in the cytoplasm and NF-κB p65 nuclear translocation. (*P < 0.01 compared with the normal group; #*P* < 0.05 compared with the DSS group and DMSO group). The blots in panel A were performed under the same experimental condition except for blotting with the different antibodies, respectively. These blots were shown as cropped images.

To determine whether an A3AR agonist modulates DSS-induced NF-κB activation, we quantitatively estimated the expression levels of IκB-α, p-IκB-α and NF-κB p65 in murine colon epithelia by western blot analysis (Fig. 4A). As expected, the expression of IκB-α was decreased and the expression of phosphorylated-IκB-α was increased in the cytoplasm of cells from DSS colitis mice (Fig. 4B, C). However, NF-κB p65 resided in the cytoplasm as an inactive form, and was absent from the nucleus (Fig. 4D) in the normal group without DSS induction. DSS colitis resulted in a shift of NF-κB p65 towards the nuclear area (increased relative to the normal group, *P* < 0.01). No significant difference was observed between vehicle-treated mice with DSS colitis and mice with DSS colitis alone. Treatment with 2-Cl-IB-MECA suppressed the phosphorylation of IκB-α (reduction of p-IκB-α in the cytoplasm compared with DSS groups, *P* < 0.05) and attenuated NF-κB p65 nuclear translocation (significant reduction in NF-κB-positive nuclei relative to the DSS groups, *P* < 0.05), which was consistent with our IF results.

### Expression of A3AR in murine colon

#### Location and qualitative expression of A3AR in the murine colon

**Figure 5:**
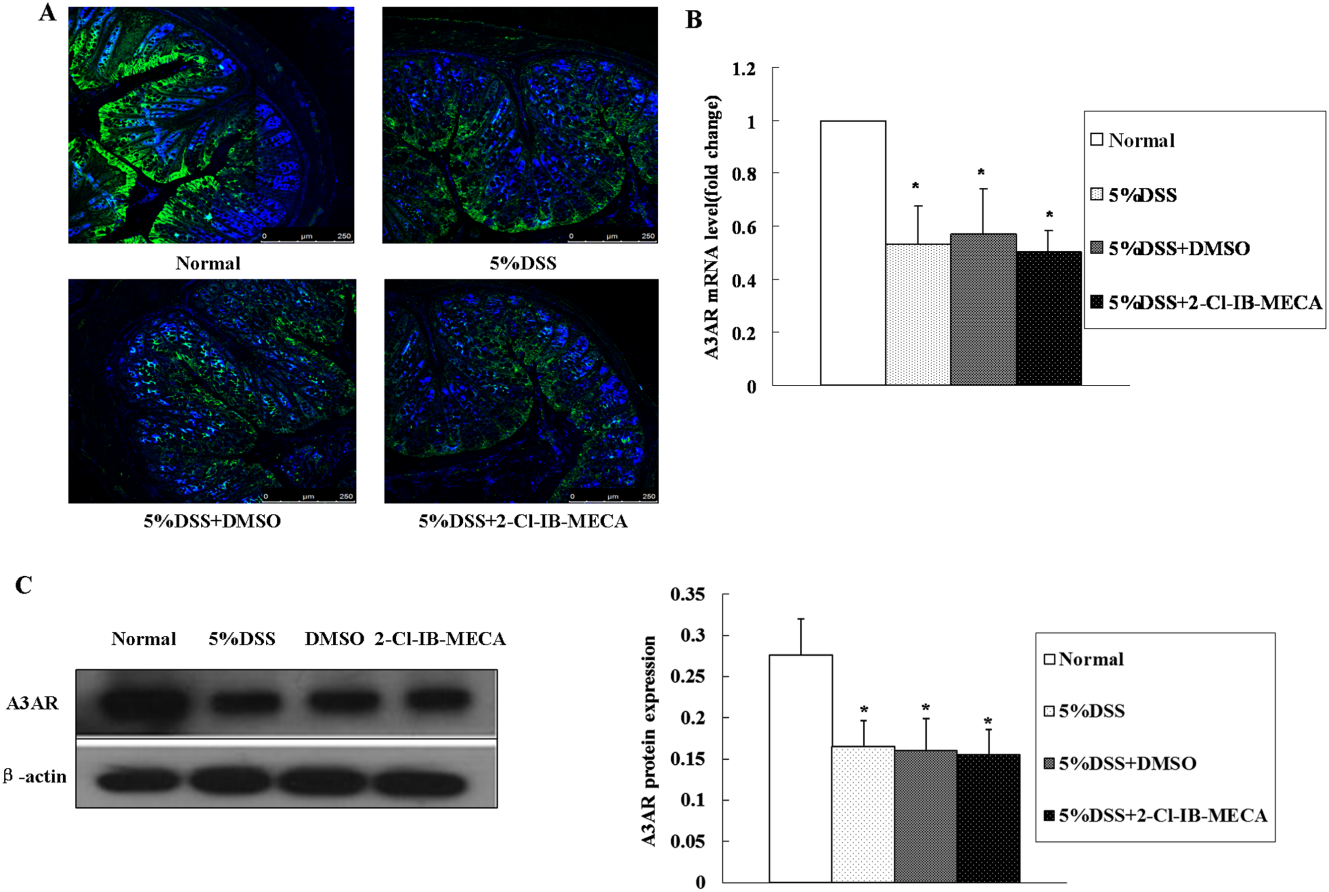
Expression of A3AR in the murine colon. (A) IF was performed using DAPI to counterstain the nuclei and a specific antibody to label A3AR proteins. The strong green fluorescence signal indicating A3AR was observed mainly in the colonic crypt epithelial cells. The expression of A3AR was reduced in DSS colitis mice. Treatment with 2-Cl-IB-MECA showed no obvious effect on A3AR expression. A3AR mRNA (B) and protein (C) expression in colonic epithelial cells were measured by qRT-PCR and western blot, respectively. Compared with the normal group, the mRNA and protein expression levels of A3AR significantly decreased after DSS induction of colitis (*P < 0.01) and showed no significant alterations among the other three groups including DSS, DMSO, and 2-Cl-IB-MECA groups (*P* > 0.05). Data are shown as mean ± SD. The blots in panel C were performed under the same experimental condition except for blotting with the different antibodies, respectively. These blots were shown as cropped images.

To verify the presence and distribution of A3AR in colon tissue, IF was performed and the micrographs are shown in Fig. 5. A3AR was observed mainly in crypt epithelial cells. The expression of A3AR in crypt epithelial cells was reduced in DSS colitis mice (Fig. 5A). Treatment with 2-Cl-IB-MECA did not alter the expression of A3AR in the colon epithelia.

### Expression of A3AR in murine colonic epithelia

The effects of DSS and 2-Cl-IB-MECA on the expression of A3AR were determined (Fig. 5B, C). Protein and mRNA expression levels of A3AR significantly decreased after DSS colitis induction, with no significant change in 2-Cl-IB-MECA-treated groups (*P* > 0.05). These data may suggest that A3AR was down-regulated after induction of DSS colitis, and 2-Cl-IB-MECA treatment did not alter the expression of A3AR in colon epithelia, consistent with our IF results.

## Discussion

Recent studies have demonstrated an important role for 2-Cl-IB-MECA in acting on A3AR with a variety of mechanisms in numerous diseases. For example, it can regulate the MEK1/2-ERK1/2 and PI3K/AKT signaling pathways in ameliorating myocardial ischemia/reperfusion injury^24^ and is involved in the extrinsic cell death pathway in human leukemia cells^25^. Activation of A3AR by 2-Cl-IB-MECA could alleviate TNF-α-induced inflammation through the inhibition of the NF-κB signaling pathway in human colonic epithelial cells^14^. However, there are no studies on the effect of 2-Cl-IB-MECA and the molecular mechanism of action of A3AR in murine colitis models in vivo. Is it the same as that observed in vitro? The interaction between 2-CL-IB-MECA and A3AR in a murine colitis model and the signaling pathways associated with this interaction remain unclear. Here we demonstrate a role for the NF-κB signaling pathway and its effect on modifying the activity of proinflammatory factors in A3AR-mediated biological processes. Our present study was performed to investigate the potential effect of the A3AR agonist 2-Cl-IB-MECA in DSS colitis mice and to elucidate the molecular mechanism involved. The primary finding from this study is that the A3AR agonist 2-Cl-IB-MECA was able to prevent the progression of gut inflammation through the NF-κB signaling pathway in a murine colitis model.

In this study, we identified A3AR expressed on colonic epithelia and demonstrated the significant reduction in mRNA and protein expression in colonic epithelia after DSS-induced colitis, which was consistent with our previous study indicating that the expression of A3AR in crypt epithelial cells was reduced significantly in DSS colitis^17^. This may be of clinical relevance since the A3AR transcript is down-regulated in mucosal biopsies from patients with Crohn's disease^26^. Moreover, we selective activated A3AR and found that administration of the A3AR agonist 2-Cl-IB-MECA alleviated the clinical symptoms and MPO activity and reduced tissue injury of DSS-induced murine colitis. However, further studies are warranted on A3AR down-regulation in the pathophysiology of colitis.

Pharmacological studies in both in vitro and in vivo models have demonstrated that activation of A3AR suppresses pro-inflammatory cytokine production through the inhibition of NF-κB activation during inflammation^14^,^27^,^28^,^29^. However, no studies have explored the effects of 2-Cl-IB-MECA-induced A3AR activation of NF-κB signaling in a DSS-induced colitis model. It is important to understand whether these effects would be the same as that observed in other models. Therefore, we aimed to investigate the relationship between A3AR and NF-κB activation in DSS-induced murine colitis model.

In agreement with the anti-inflammatory role of A3AR, our studies found that 2-Cl-IB-MECA attenuated DSS colitis-induced NF-κB activation in murine colonic epithelia. Several lines of evidence suggest that NF-κB is a key modulator governing the molecular network leading to various cellular functions associated with IBD^3^. Besides, it is now well known that NF-κB is an inducible transcription factor that mediates signal transduction between the cytoplasm and the nucleus in many cell types, including intestinal epithelial cells^1^,^30^. NF-κB exists in the cytoplasm as an inactive form, and its activation is tightly regulated by IκB-α, which undergoes phosphorylation and degradation, followed by NF-κB nuclear translocation^31^,^32^. As evaluated by IF and western blot analyses in this study, DSS-induction of colitis resulted in rapid phosphorylation and degradation of IκB-α in colonic epithelia, and the obvious nuclear translocation of p65 can be observed. In contrast, the A3AR agonist 2-Cl-IB-MECA inhibited DSS colitis-induced phosphorylation of IκB-α and the nuclear translocation of NF-κB p65 in colonic epithelia. These results indicate that 2-Cl-IB-MECA has a role in the suppression of DSS colitis-induced NF-κB activation. It is therefore conceivable that A3AR activation was involved in DSS-induced colitis through the modulation of the NF-κB pathway in colonic epithelia.

In addition, 2-Cl-IB-MECA mediated a reduction of the expression of pro-inflammatory cytokines TNF-a and IL-1β. Upon induction of DSS-colitis, NF-κB was activated and translocated to the nucleus of colonic epithelial cells^33^,^34^, where it regulated the transcription of a series of genes. In our study, we found that the mRNA levels of TNF-a and IL-1β increased in the DSS colitis mice. Interestingly, this up-regulation was inhibited by treatment with 2-Cl-IB-MECA. These data suggest that 2-Cl-IB-MECA can inhibit the expression of TNF-a and IL-1β in colonic epithelia of DSS colitis mice.

In the present study, 2-Cl-IB-MECA significantly inhibited the NF-κB pathway in the colonic epithelia of DSS colitis mice. Inhibition of both NF-κB activation and IκBa phosphorylation was associated with the suppression of pro-inflammatory cytokines expression in colonic epithelia of DSS colitis mice. 2-Cl-IB-MECA also attenuated acute murine colitis induced by DSS administration. To the best of our knowledge, this is the first mechanistic determination of the anti-inflammatory effect of 2-Cl-IB-MECA in a murine DSS colitis model. Further studies are required to understand whether other molecular mechanisms are involved in the effects of 2-Cl-IB-MECA in the development of DSS colitis. This will help to clarify the anti-inflammatory mechanisms of A3AR activation, which were consistent with those described in previous studies^16^,^35^.

On the contrary, Ren T et al., by using female mice (3–4 months or 8–10 months of age) in a two-week DSS-induced colitis model, found that A3−/−AR protects against DSS-colitis. This was consistent with a novel hypothesis that A3AR activation contributes to the development of colitis^17^. It indicated that A3AR has both anti- and pro-inflammatory effects in DSS-induced colitis, and this effect might be associated with mice from different developmental stages, gender, the time of DSS induction or gene expression, and so on.

In conclusion, our findings indicate that A3AR is expressed in colonic epithelia and its selective activation has an anti-inflammatory activity through the inhibition of pro-inflammatory cytokine expression associated with the inhibition of NF-κB signaling pathways in murine DSS colitis in vivo. Therefore, the protective effects of 2-Cl-IB-MECA on murine colitis support its potential usefulness for the treatment in patients with IBD.

## Methods

### Induction of murine colitis and administration of 2-Cl-IB-MECA

Thirty specific pathogen-free mice (BALB/C male mice; age, 8–10 weeks; weight, 18–22 g) were breed from the Experimental Animal Center of Guangdong Medical College, Guangdong, China. All animal experiments were approved by the Animal Care Committee of Guangdong Medical College and performed in accordance with relevant guidelines and regulations. Mice were group-housed (a maximum of four mice/cage) under a controlled temperature (25°C) and a 12:12-h light-dark cycle and were allowed free access to standard mouse chow and water. DSS-water consumption, food intake, and body weight of the animals were monitored and recorded on a daily basis. Colitis in mice was induced by the administration of 5% DSS (molecular weight, 40,000–50,000; MP Biomedicals, Irvine, CA, USA) in drinking water for 7 days. The drinking water was then replaced with normal saline solution for another 7 days. The selective A3AR agonist 2-Cl-IB-MECA (2-Cl-IB-MECA group) (1.0 mg/kg/day in 0.2 mL) (Tocris Bioscience, United Kingdom) or 37.5% dimethyl sulfoxide (DMSO) (vehicle group) was administered to the mice intragastrically by a feeding needle once a day, starting at day 1 from colitis induction. The DSS group received a gavage of normal saline solution instead of the drug, while the control group was administered sterile drinking water for 14 days and received a gavage of sterile water instead of the drug.

The mice were divided into four groups. In the first group, designated the normal control group (n = 6), colitis was not induced and no therapeutic intervention was performed. The second group, designated the DSS group (n = 8), received 5% DSS and normal saline solution orally. The third group, designated the vehicle group (n = 8), received 5% DSS and DMSO. The fourth group, designated the 2-Cl-IB-MECA group (n = 8), received 5% DSS and 2-Cl-IB-MECA (1.0 mg/kg/day) dissolved in DMSO. Typically, the experiments were carried out for 2 weeks, and on the 14th day after the induction of colitis. All experiments were performed under gaseous anesthesia and animals were euthanized by overdosage of anesthesia followed by cervical dislocation. The colon was removed and processed for histopathology or biochemical studies.

### Assessment of disease severity in DSS colitis mice

#### Clinical activity evaluation of weight loss, stool consistency (diarrhea), and guaiac test of fecal occult blood

The guaiac test (Baso Diagnostics, Inc. Zhuhai, China) was used to detect fecal occult blood in the gastrointestinal tract, which is indicative of gastrointestinal bleeding associated with severe intestinal inflammation and tissue damage in the DSS colitis model. Fecal pellets from each mouse were tested for a positive guaiac test every day during the 2-week study, and data were analyzed in terms of the number of animals with a positive test result on each day.

### Histopathological assessment of the colon in DSS colitis mice

The distal colon was removed and then fixed overnight in 4% (w/v) paraformaldehyde in PBS. Paraffin sections (5-μm sections, transverse orientation) were processed for histopathological scoring. The histological score of haematoxylin and eosin (H&E)-stained sections of the colon was determined by a pathologist in a blinded fashion, according to a previously described method^17^.

Histopathological scoring was performed separately for inflammation, crypt damage, and ulceration^36^. Briefly, for inflammation, score 0: rare inflammatory cells in the lamina propria; score 1: increased numbers of granulocytes in the lamina propria; score 2: confluence of inflammatory cells extending into the submucosa; score 3: transmural extension of the infiltrate. For crypt damage, score 0: an intact crypt; score 1: loss of the basal one-third of the crypt; score 2: loss of the basal two-thirds of the crypt; score 3: entire crypt loss; score 4: change of epithelial surface with erosion; score 5: confluent erosion. For ulceration, score 0: absence of ulcer, score 1: 1 or 2 foci of ulcerations; score 2: 3 or 4 foci of ulcerations; score 3: confluent or extensive ulceration. The aggregate score of the individual histopathological scores ranged from 0 to 11. For statistical analysis, we averaged the aggregate scores of the sections for each colon.

### Myeloperoxidase (MPO) activity

Colonic MPO activity was used as an index of neutrophil infiltration into the injured/inflamed mucosa. Briefly, MPO activity was measured with an MPO enzyme-linked immunosorbent assay (ELISA) kit (Sangon Biotech, Shanghai, China). The colonic samples (20 mg, after removing any visible feces or fat by using bent forceps) were thawed and homogenized on ice in 0.5 mL PBS buffer. The subsequent assay was performed according to the manufacturer's instructions. All experiments were performed in triplicate. Concentrations of MPO in the colon were expressed as ng/mL.

### Immunofluorescence (IF) analysis of A3AR, IκB-α, phosphorylated-IκB-α, and NF-κB p65

IF staining was performed according to standard protocols by using 5-μm thick transverse-cut paraffin sections from colons of control or DSS colitis mice. Briefly, paraffin sections were rendered permeable by incubation in 0.1% Triton X-100 for 30 min; the sections were then blocked with 10% donkey serum albumin at room temperature (RT) for 30 min and incubated with primary antibodies (Santa Cruz Biotechnology, Dallas, TX, USA) against A3AR (1:30 dilution), NF-κB p65 (1:50 dilution), nuclear factor-kappaB inhibitor alpha(IκB-α) (1:50 dilution), or phosphorylated-IκB-α (p-IκB-α) (1:50 dilution) overnight at 4°C. After rinsing three times with phosphate buffered saline (PBS), sections were incubated with secondary antibodies (Donkey anti-rabbit FITC, Donkey anti-goat DyLight™405, Donkey anti-mouse Cy™3, 1:100 dilution, Santa Cruz Biotechnology) for 1 h at room temperature in dark, then washed three times with PBS. Negative controls were prepared by omitting the primary antibodies. Finally, coverslips were mounted on slides by using fluorescent mounting medium with DAPI to counter-stain the nuclei. The staining was evaluated on a Leica inverted fluorescence microscope.

### Isolation of colonic epithelial cells and extraction of cytosolic and nuclear protein

The isolation of colonic epithelial cells was performed on the basis of previously described procedures^37^. Cytoplasmic or nuclear extracts from colon epithelia were separately collected using a Sangon Biotech Kit (Shanghai, China), according to the manufacturer's protocols. The protein concentrations in cytoplasmic or nuclear lysates were determined using a bicinchoninic acid (BCA) protein assay kit (Sangon Biotech, Shanghai, China).

### Western blot analysis of A3AR, IκB-α, p-IκB-α, and NF-κB p65

Equal amounts of protein (20–30 μg) were separated by 10% SDS-polyacrylamide gel electrophoresis (SDS-PAGE) and transferred to a polyvinylidene fluoride membrane (PVDF, Millipore, Billerica, MA, USA). The membrane was blocked for 1 h with 5% fat-free dried milk at RT, then incubated with primary antibodies (Santa Cruz Biotechnology) against A3AR (1:800 dilution), NF-κB p65 (1:600 dilution), IκB-α (1:400 dilution), p-IκB-α (1:400 dilution), Tubulin (1:1,000 dilution), or β-actin (1:1,000 dilution) at 4°C overnight. Membranes were washed three times with Tris-buffered saline with Tween-20 (TBS-T) and incubated with the corresponding secondary antibodies (HRP-labeled Goat Anti-mouse IgG, HRP-labeled Goat Anti-rabbit IgG, HRP-labeled Donkey Anti-goat IgG; 1:1,000; Beyotime, China) for 1 h at room temperature. Signals were detected with an electrochemiluminescence (ECL) detection reagent (Beyotime, China). The images were obtained on Kodak film and quantified by Quantity One software (Bio-Rad, Hercules, CA, USA).

### Real-time Quantitative Reverse Transcription Polymerase Chain Reaction Analysis (qRT-PCR) of A3AR and proinflammatory cytokines TNF-α and IL-1β mRNA expression

For qRT-PCR experiments, total RNA from colon epithelia was extracted using RNAiso Plus by following the manufacturer's instructions (Takara, Japan). Total RNA (500 ng) was converted to cDNA, and PCR reactions were performed in triplicate using a SYBR Green PCR LightCycler® (Roche Diagnostics, Indianapolis, Indiana, USA) in a 96-well format over 45 cycles with denaturation at 95°C for 10 s and annealing at 58°C for 20 s. The primers for mouse β-actin, A3AR, TNF-a, and IL-1β were purchased from Sangon Biotech (Shanghai, China). The sequences of these primers were listed in Table 1. Data were normalized versus β-actin for mouse A3AR, TNF-a, and IL-1β by using a 2^−ΔΔCT^ method. Data were expressed as the fold change in mRNA transcript levels relative to that of the normal control.

### Statistical analysis

Data are expressed as mean ± standard deviation (SD). Multiple groups were compared by a one-way analysis of variance (ANOVA) and post-hoc tests between individual groups. SPSS 16.0 software (SPSS Inc. USA) was used for analyses. Histopathology was analyzed by a nonparametric Mann–Whitney U-test. The chi-square test was used to analyze data from the guaiac test for the presence of occult blood in the stools or stool consistency (diarrhea). Differences were considered statistically significant at *P* < 0.05.

## Author Contributions

Y.Z. conceived the study. T.H.R., X.F. and Y.Z. designed the experiments. T.H.R., T.T., X.F., S.C.Y., H.W., W.Y.W., Y.M.Q., C.Y.Y., Y.T.H., J.C.Z., J.W.C. and Y.Z. performed the experiments. T.H.R., T.T. and X.F. analyzed the data. T.H.R., T.T., X.F. and Y.Z. wrote the paper.

## Figures and Tables

**Table 1:** Primers used for quantitative reverse transcription-polymerase chain reaction in this study

Name	Direction	Primer (5′-3′)
β-actin	Forward	CTTCTTCTTGGTATGGAATCCTG
	Reverse	GTAATCTCCTTCTGGATCCTGTC
A3AR	Forward	CGGGAGTTCAAGACAGCTAAGT
	Reverse	CACATTGCGACATCTGGTATCT
TNF-a	Forward	ACGGCATGGATCTCAAAGAC
	Reverse	GTGATCTCCTTCTGGATCCTGTC
IL-1β	Forward	CAGGCAGGCAGTATCACTCA
	Reverse	TGTCCTCATCCTGGAAGGTC
